# Supplementary material for: Nonadditivity in Many-Body Interactions between Membrane-Deforming Spheres Increases Disorder
Source: ACS Nano. 2024 Aug 15;18(34):23067–76. doi: 10.1021/acsnano.4c05222 (PMC11363220; doi:10.1021/acsnano.4c05222)
Supplement: Supplementary file 14 — nn4c05222_si_014.pdf [file nn4c05222_si_014.pdf]

Supporting Information

. Non-additivity in many-body interactions between membrane-deforming spheres increases disorder

Ali Azadbakht[1], Thomas R. Weikl[2], and Daniela J. Kraft[1]

[1]Soft Matter Physics, Huygens-Kamerlingh Onnes Laboratory, Leiden University, PO Box 9504, 2300 RA Leiden, the Netherlands
[2]Department of Biomolecular Systems, Max Planck Institute of Colloids and Interfaces, Am Mühlenberg 1, 14476 Potsdam, Germany

Movie S1: Video of the experimental observation by confocal microscopy of the attraction between two membrane-deforming colloidal spheres pulled underneath a GUV with two optical traps.

Movie S2: Video of experimental force measurement between two particles with optical tweezers. The spherical particle on the left is placed in a trap with a fixed position and the force exerted on it is measured using the displacements from its equilibrium position. The right particle is moved to vary the distance between the particles.

Movie S3: Confocal microscope video of the observation of the attraction of three membrane-deforming colloidal spheres after they were pulled under a GUV and initially held in line in three optical traps. After the optical traps were turned off, the particles first decreased the distance and then formed a compact triangular cluster.

Movie S4: Video of experimental force measurement between three membrane deforming particles taken by confocal microscopy. Two particles are held in two moving traps and form a "dumbbell". The single particle is held in a fixed trap as a force sensor at different distances and angles with respect to the dumbbell.

Movie S5: Experimental observation by confocal microscopy of four particles underneath of a GUV showing a rearrangement between two compact diamond states.

Movie S6: Video showing the local bond orientational order parameter Ψ_6 in time, where 1 corresponds to the perfect hexagonal ordering for 9 membrane-deforming particles.

Movie S7: Video showing the local bond orientational order parameter Ψ_6 in time, where 1 corresponds to the perfect hexagonal ordering for 11 membrane-deforming particles.

Movie S8: Video showing the local bond orientational order parameter Ψ_6 in time, where 1 corresponds to the perfect hexagonal ordering for 24 membrane-deforming particles.

Movie S9: Video showing the local bond orientational order parameter Ψ_6 in time, where 1 corresponds to the perfect hexagonal ordering for 36 membrane-deforming particles.

Movie S10: Video showing of the number of nearest neighbors in time for 9 particles confined underneath a GUV obtained by Voronoi tessellation, where tiles of (5) neighbors are colored green, (6) white, (7) red, others in blue.

Movie S11: Video showing the number of nearest neighbors in time for 11 particles confined underneath a GUV obtained by Voronoi tessellation, where tiles of (5) neighbors are colored green, (6) white, (7) red, others in blue.

Movie S12: Video showing the number of nearest neighbors in time for 24 particles confined underneath a GUV obtained by Voronoi tessellation, where tiles of (5) neighbors are colored green, (6) white, (7) red, others in blue.

Movie S13: Video showing the number of nearest neighbors in time for 36 particles confined underneath a GUV obtained by Voronoi tessellation, where tiles of (5) neighbors are colored green, (6) white, (7) red, others in blue.

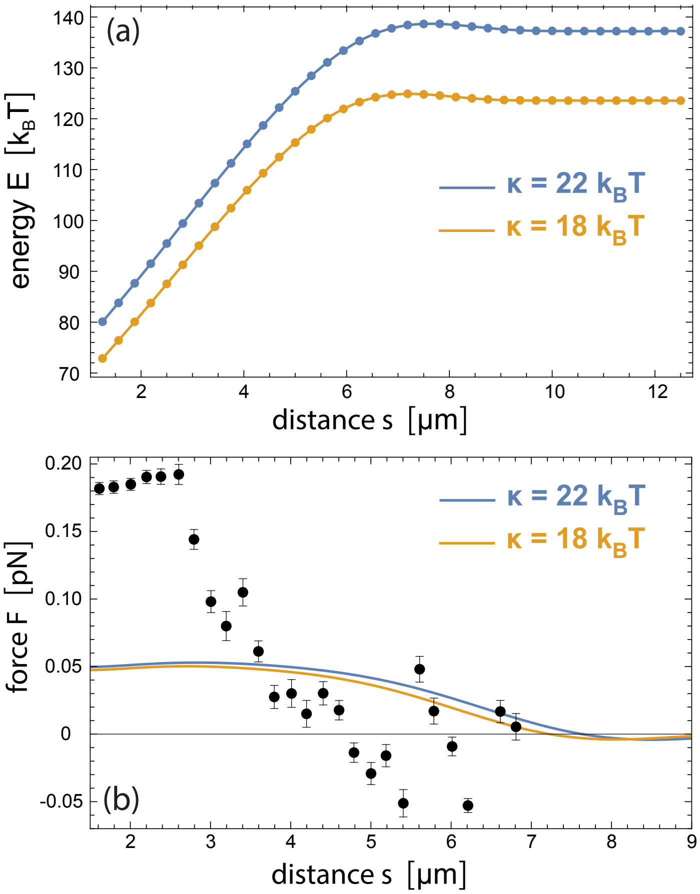

Figure S1: **Modeling results under the assumption of constant gravitational pressure compared to experimental results.** (a) Total energy $E = G + pV$ versus distance s of two particles from energy minimization at the constant gravitational pressure $p = 1.7\, k_BT/\mu m^3$ resulting from the density difference $\Delta\rho = 22.6\,kg/m^3$ between inner and outer vesicle solution (see Results section) and the vesicle height $h_v = 32\,\mu m$ in the experiments of Fig. 1. Here, G denotes the bending energy, and V denotes the total volume of the membrane-covered solvent pocket(s) of the two particles (see Methods). The modeling has been conducted at the two exemplary bending rigidites $\kappa = 22\ k_BT$ (blue line and data points) and 18 k_BT (yellow line and data points). As in Fig. 1, the lines represent 12th order polynomial fits of the minimization results (points). (b) Comparison of the measured forces of Fig. 1(g) (data points) to calculated forces (lines) obtained as derivatives of the energy curves in (a). The modeling curves under assumption of constant gravitational pressure here clearly deviate from the experimentally measured forces, which confirms the modeling assumption of constant confinement volume of the particles in Fig. 1. In addition, the volume $v = V/2$ per particle resulting from the energy minimizations at constant gravitational pressure p is $v = 19.4\,\mu m^3$ and $v = 17.4\,\mu m^3$ for large particle distance and the membrane bending rigidities $\kappa = 22\ k_BT$ and 18 k_BT, respectively, which is much larger than the volume $v = 7.8 \pm 1.6 \mu m^3$ per particle obtained from experiments (see Fig. S2). At particle contact, the volume v per particle obtained at constant gravitational pressure p is $12.5\,\mu m^3$ and $11.3\,\mu m^3$, respectively.

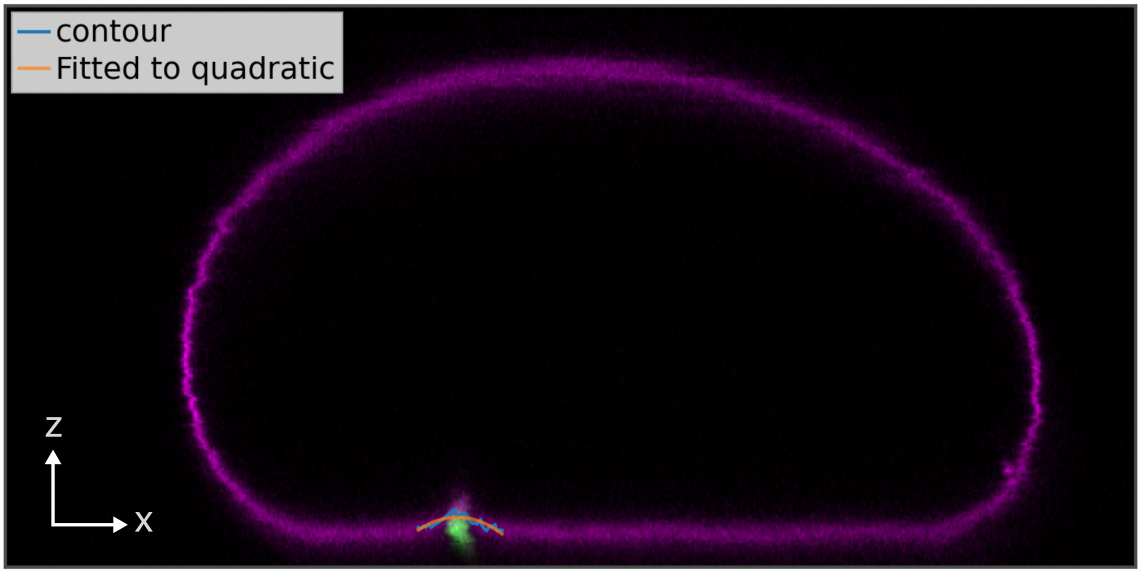

Figure S2: **Example of a confocal image of the zx plane of GUV (magenta) and membrane-deforming sphere (green).** The membrane contour in the vicinity of the sphere (blue line) was fitted to a quadratic polynomial equation (orange line). The fitted curve was revolved to measure the volume around the distorted and flattened membrane.

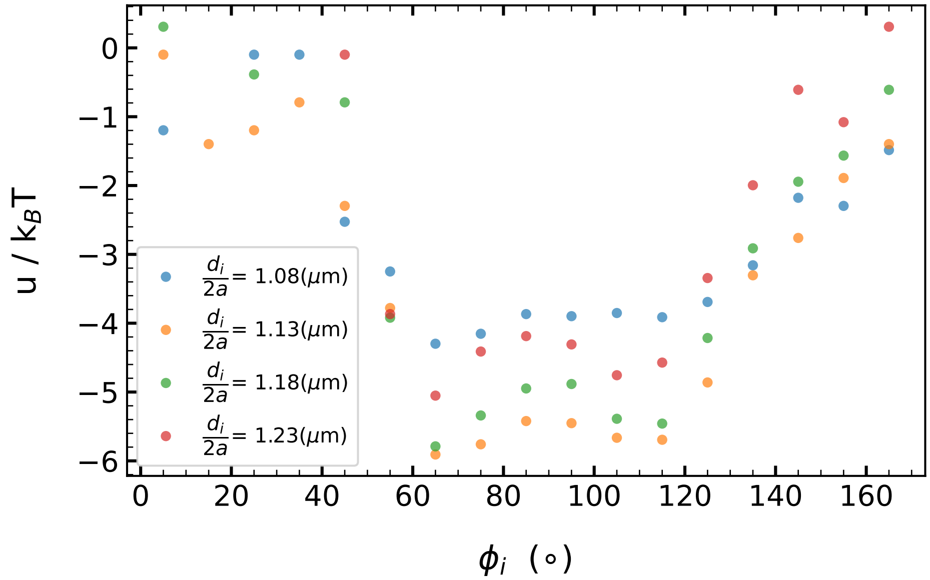

Figure S3: **Interaction energy** ($\frac{u}{k_B T}$) for four membrane deforming spheres as a function of ϕ_i for different values of $d_i/2a$. The data is the same as presented in Figure 4 in the main text, only plotted as line profiles for fixed values of $d_i/2a$.

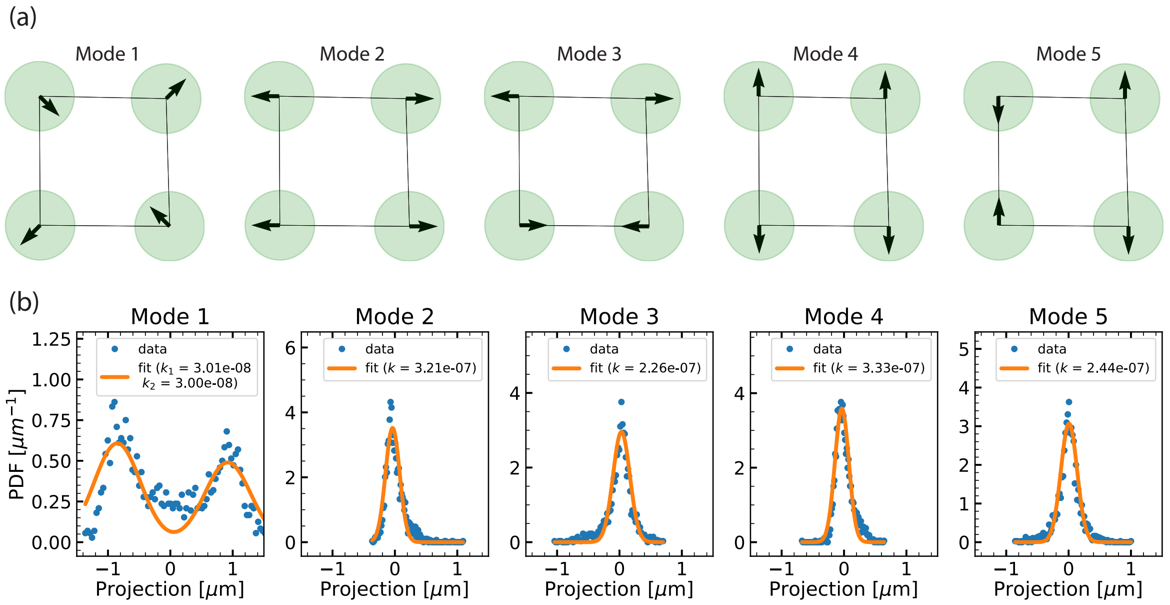

Figure S4: **Mode analysis for four membrane-deforming particles:** (a) Schematics showing modes present in the system; (b) Frequency of finding the modes as a function of projection distance.

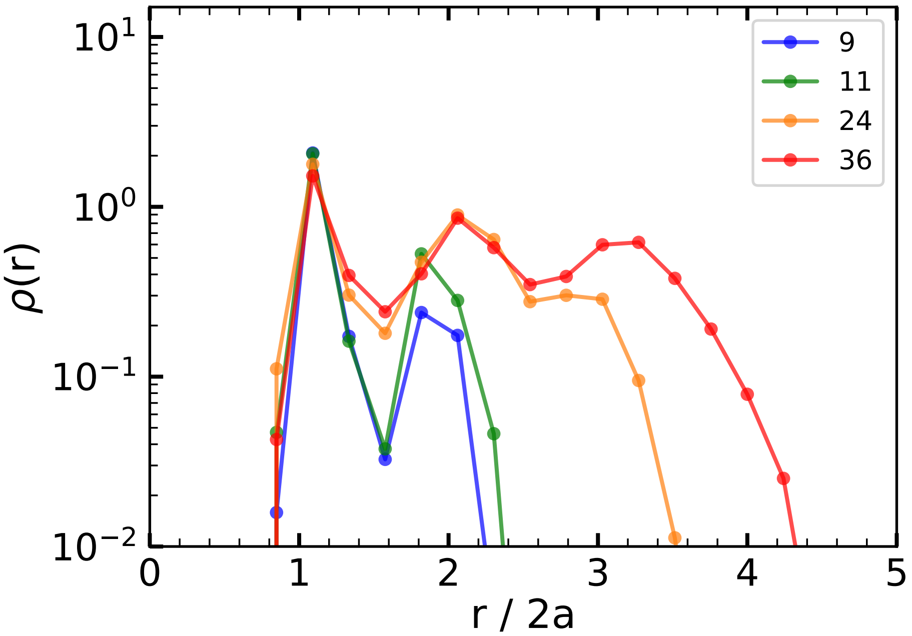

Figure S5: **Radial density profile** $\rho(r)$ as a function of rescaled distance $r/2a$ where a is the radius of the particles, in a semi-logarithmic scale for different numbers of particles confined underneath a GUV as indicated in the legend.

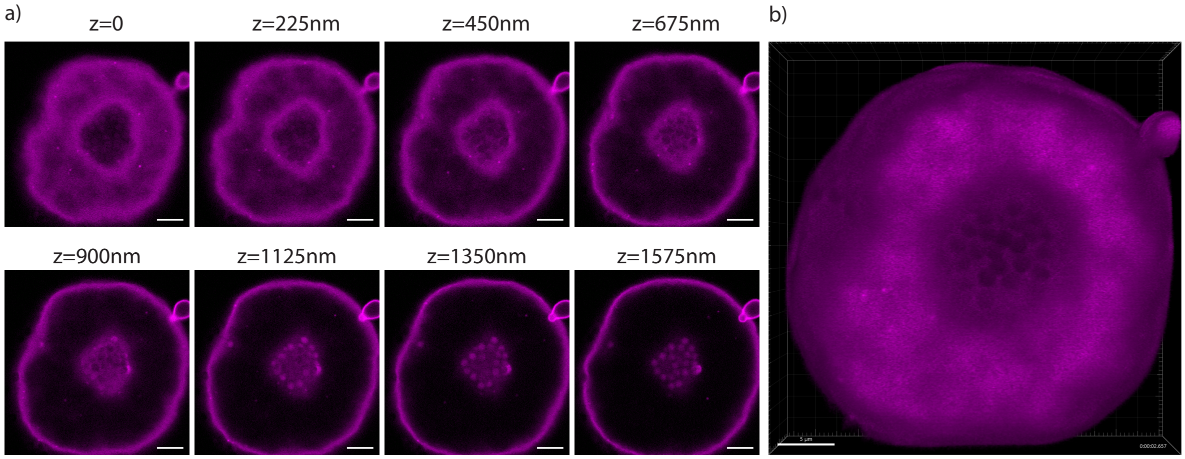

Figure S6: **Confocal observation of a GUV deformed by 24 non-fluorescent particles.** To mitigate cross-talk between fluorescent channels, we utilized non-fluorescent colloids, confirming their interaction through the membrane-deformation. a) Z-stacks of confocal images taken at different heights above the glass substrate. b) Reconstructed 3D image of the GUV.
